# Metal-Containing Proteins, Macrocycles, and Coordination Complexes in Therapeutic Applications and Disease

**DOI:** 10.1155/2008/286363

**Published:** 2008-09-11

**Authors:** Jannie C. Swarts, Michael J. Cook, Edward N. Baker

**Affiliations:** ^1^Department of Chemistry, University of the Free State, Bloemfontein 9300, South Africa; ^2^School of Chemical Sciences and Pharmacy, University of East Anglia, Norwich NR4 7TJ, UK; ^3^Centre for Molecular Biodiscovery and School of Biological Sciences, University of Auckland, 3A Symonds Street, Private Bag 92019, Auckland 1142, New Zealand

Treatment of diseases with natural and synthetic materials has been an aspiration of mankind since the dawn of human development. From the use of willow-bark to the marketing of aspirin, a steady move from folk remedies to the use of chemistry and biology to develop new therapies has been observed. In terms of metal-containing drugs, the platinum-containing drug cisplatin has long been the most effective metal-containing anticancer drug on the market.

However, severe side effects of conventional drugs are associated with the inability to distinguish between healthy and cancer cells. Hence, a concerted world-wide effort is in progress to discover and characterise new drugs that may distinguish between healthy and cancer or other diseased cells. New techniques of drug delivery are sought and the use of natural products, proteins, antibodies, and synthetic polymers as drug delivery devices capable of targeting a diseased site is being investigated.

These issues are nicely illustrated by macrocycles such as porphyrins, phthalocyanines, and related systems. Some of these compounds exhibit selective absorption by cancer cells and have the ability to photosensitize formation of singlet oxygen. These attributes have led to the development of alternative cancer treatments known as photodynamic therapy. Sadly, many potentially good new therapeutic agents often never leave the designers' laboratory due to some pharmacological problems associated with its in vivo use. The use of drug delivering devices, including water-soluble synthetic polymeric drug delivery systems, may help overcome many pharmacological drug-related problems, including those of solubility, specificity, and biocompatibility, factors that currently prevent many potentially good therapeutic agents from reaching clinics.

The focus of this special issue is the synthesis, characterisation, physical studies, and application of synthetic metal-containing complexes and natural occurring proteins in serious human diseases such as cancer, diabetes, arthritis, viral disease, malaria, and tuberculosis with special focus on the following:

- porphyrins, phthalocyanines, and related complexes in photodynamic cancer therapy;
- proteins, enzymes, and synthetic polymeric drug delivery systems in the treatment of cancer and other diseases;
- coordination and organometallic compounds in cancer, arthritis, malaria, and viral disease.

Towards these goals, L. Josefsen and R. Boyle describe in their review article the development and application of metal-based photosensitisers, including porphyrins and phthalocyanines, in photodynamic therapy. Four other publications highlight different aspects of porphyrin-based macrocyclic photosensitisers. S. Lee et al. focus on the cellular uptake and toxicity of thiotetra (ethylene glycol) monomethyl ether-functionalized porphyrazines. J.-Y. Liu et al. focus on in vitro photodynamic activity of novel amphiphilic zinc(II) phthalocyanines bearing oxyethylene-rich substituents. E. Antunes and T. Nyokong highlight the syntheses and photophysical properties of tetraazatetrabenzcorrole photosensitizers. Sakamoto et al. present a fundamental study of zinc bis(1,4-didecylbenzo)-bis(2,3-pyrido)porphyrazine for application in photodynamic therapy of cancer.

Considering polymeric drug delivery systems, South African E. Neuse's excellent review describes the use of synthetic polymers as metal-containing drug delivery vehicles in medicine. M. David Maree et al. provided a fine treatise on why biocompatible synthetic polymeric drug delivery systems are becoming increasing popular as drug delivering devices. They also demonstrate the principles behind these systems in a practical study utilising ferrocene and phthalocyanine derivatives anchored on a water-soluble polymeric drug carrier derived from lysine and aspartic acid. Italians Longo and Vasapollo demonstrated the use of phthalocyanine-based molecularly imprinted polymers as nucleoside receptors. X. Sun et al. report on the identification of proteins related to nickel homeostasis in *Helicobater pylori* by immobilized metal affinity chromatography and two-dimensional gel electrophoresis. P. Nagababu reported DNA-binding and photocleavage studies of cobalt (III) ethylenediamine pyridine complexes.

M. Blackie and K. Chibale's excellent minireview focuses on metallocene antimalarials. The development of new metal-containing chemotherapeutic drugs is highlighted by contributions from M. Hogan et al. in their synthetic and cytotoxic study of new titanocene analogous. S. Mahepal et al. look at the in vitro antitumor activity of novel, mitochondrial-interactive, gold-based lipophilic cations. Sathisha et al. describe bis-isatin thiocarbohydrazone metal complexes and their antitumor activity against Ehrlich Ascites Carcinoma in Swiss albino mice. In conclusion, J. M. Botha and A. Roodt report mechanistic studies on trans-aquatetracyanooxo complexes of Re(V) and Tc(V) and discuss the implications of their results for nuclear medicine.

We hope that this special issue will stimulate new research in all areas of metal-containing drug research and that it will help to focus research efforts of new and experienced researchers on key problems in the exciting field of metal-containing drug research.

*Jannie C. Swarts* *Jannie C. Swarts*

*Michael J. Cook* *Michael J. Cook*

*Edward N. Baker* *Edward N. Baker*

